# Strengthening Animal-Human Relationships as a Doorway to Indigenous Holistic Wellness

**DOI:** 10.1089/eco.2019.0003

**Published:** 2019-08-30

**Authors:** Angela McGinnis, Adela Tesarek Kincaid, M.J. Barrett, Corinne Ham

**Affiliations:** ^1^ University of Regina, Regina, Canada.; ^2^ University of Saskatchewan, Saskatoon, Canada.; ^3^ Beardy's-Okemasis First Nation, Canada.

**Keywords:** Animals, Indigenous health, Holistic wellness, More-than-human, Reconciliation, Storytelling

## Abstract

*One of the most devastating effects of colonization has been fragmented relations among humans and their more-than-human counterparts. Traditionally, Indigenous peoples positioned animals as equitable partners in interconnected human and more-than human networks, animated with spirit and the ability to act and communicate. Many Indigenous peoples continue to regard animals as sacred and utilize the gifts that they bestow in traditional healing settings. Indigenous understandings of interwoven and reciprocal social networks of human and more-than-human relations must be restored and supported in contemporary health settings in order to “do no further harm” and facilitate Indigenous peoples' healing journeys. Reconciliation across Western and Indigenous contexts requires learning to work together with the more-than-human world and developing ethical spaces for health research in which holistic wellness is appreciated and understood in the context of* all our relations. *In order to help (re)connect and strengthen human relations with the more-than-human world, a culturally adapted and locally refined animal-human relationship workshop was delivered in a rural Saskatchewan First Nation community where traditional Elders, adults, and youth participants shared stories about the role of animals for their healing and holistic wellness trajectories. The results revealed that animal-human relationships are physical and spiritual in nature, with both domestic and wild animals playing various important person roles in the lives of community members; these person roles are not metaphorical but rather assume all the sentience and agency that the term* person *implies. The findings have clear practical and policy implications for health services, education, environmental sustainability, and bioresource management.*

## Introduction

The Indigenous concept of *all our relations* highlights the integral interconnections between humans and the more-than-human world for Indigenous peoples. Traditionally, Indigenous peoples positioned animals as equitable partners in an interconnected social network of human and more-than-human beings, animated with spirit and the ability to act and communicate (Legge & Robinson, 2017). Strong and healthy relationships between human and more-than-human beings have long been understood by Indigenous peoples as critical to achieve *mino-bimaadiziwin,* the way of a good life or living life in its fullest sense, free from illness and misfortune (Hallowell, 1960; Rheault, 1999). Unfortunately, one of the most devastating effects of colonization has been fragmented relations among humans and their more-than-human counterparts (Kimmerer, 2013; LaDuke, 1999/2015; Tuhiwai-Smith, 1999). Despite these cultural losses, many Indigenous peoples today continue to regard animals as sacred and utilize the gifts that they bestow in traditional healing settings (Hallowell, 1960; Matamonasa-Bennett, 2015; Nadasdy, 2007). However, contemporary health care services fail to build on these interconnections as a core strength of Indigenous cultures. Instead, Western treatment models tend to focus on the “individuated being” (Latimer & Miele, 2013, p. 8), which overlooks or dismisses the natural healing resources embedded in Indigenous relationships with the land and all of its inhabitants (i.e., two-leggeds, four-leggeds, swimmers, crawlers, and winged-ones in both physical and spiritual forms; Fenelon & LeBeau, 2006; Kawagley, 2001).

While research-informed efforts to adapt evidence-based health promotion and treatment approaches for cultural relevance are welcomed and needed, much research in this area continues to be driven by deficit-based approaches that fail to acknowledge and consider the role of more-than-human beings in the lives of Indigenous peoples (Crooks, Snowshoe, Chiodo, & Brunette-Debassige, 2013; Mohatt, Fok, Burket, Henry, & Allen, 2011). Although these initiatives may be well intentioned, the lack of attention paid to the role of these relationships results in a “silencing” of the more-than-human world that subsequently serves to undermine and trivialize the very healing resources that support wellness in Indigenous contexts (Castellano, 2015; LaFromboise, Trimble, & Mohatt, 1990; Mussell, Cardiff, & White, 2004). Fully understanding the unique health needs of Indigenous peoples requires strengths-based approaches that focus on (re)connecting individuals, families, and communities to a cultural way of being that is grounded in a more-than-human worldview (Duran & Duran, 1996; McCormick, 1998; Snowshoe, Crooks, Tremblay, & Hinson, 2017). A significant paradigm shift is needed to align health promotion approaches with Indigenous epistemologies, ontologies, and methodologies where an all-inclusive relationship with the world is positioned as pertinent and central to Indigenous peoples' holistic health (Greenwood, de Leeuw, Lindsay, & Reading, 2015; Kirmayer, Tait, & Simpson, 2009; Snowshoe et al., 2017).

Acknowledging the gaps in past research and practice, our research team worked closely with community Elders, adults, health and education providers, youth, and animals to (a) support the process of remembering and sharing community stories of animal-human relationships that have been weakened in the processes of colonization; (b) revitalize those relationships by (re)learning practices to communicate physically and spiritually with animals in culturally-specific ways; (c) capture community-derived meanings from those strengthened relationships; and (d) synthesize those meanings to create a mutually beneficial Indigenous wellness model for humans and animals alike that is grounded in the concept of *all our relations* (Adams et al., 2015; Argent, 2012; Armenta, Whitbeck, & Habecker, 2016; Baker & Baker, 2010; Battiste, 2013). Together, we investigated the health-related impacts of delivering a culturally adapted and locally refined animal-human relationship workshop in the community and co-created an Indigenous wellness model that supports humans and animals as therapeutic allies.

## Indigenous Methodology and Research Approach

It has been well documented that Indigenous research must be designed and conducted in close collaboration with community members during all stages of the research process to be effective and ethical (Castellano, 2004, 2000; Ermine, 2007; Ermine, Sinclair, & Browne, 2005; Flicker et al., 2015). Furthermore, the process of conducting research itself needs to be decolonized in ways that take into account Indigenous epistemologies, ontologies, and community-based ethics and cultural protocols (Hart, 2010; Tuhiwai-Smith, 1999; Wilson, 2008). This initiative was built upon a foundation of culturally meaningful and lasting community-based research partnerships, an expressed community need, its potential to immediately address this need, and its long-term potential to develop an Indigenous wellness model grounded in the wishes, desires, and wisdom of the traditional Elders from the community. Both formal and moral cultural-political approval and support were acquired from the Chief and Band Council of the community and from the traditional Elders' Council, respectively. Joint research ethics approval was received from the two participating university research ethics boards. Spiritual support was requested through an initial offering of cloth and tobacco followed by a sweatlodge ceremony with the research team and community stakeholders, which was continually renewed as well as ongoing prayers at key points throughout our collective process. Each research team member's own individual spiritual practices were also central to our relational approach, which centered both human and more-than-human relations. Our community-based research partnership situated community researchers (two people), university researchers (seven people), traditional Elders (six people), adults (nine people), and youth (seven people) as both experts and learners simultaneously, a dynamic that was critical to the start and overall success of the research project.

**Fig. 1.**
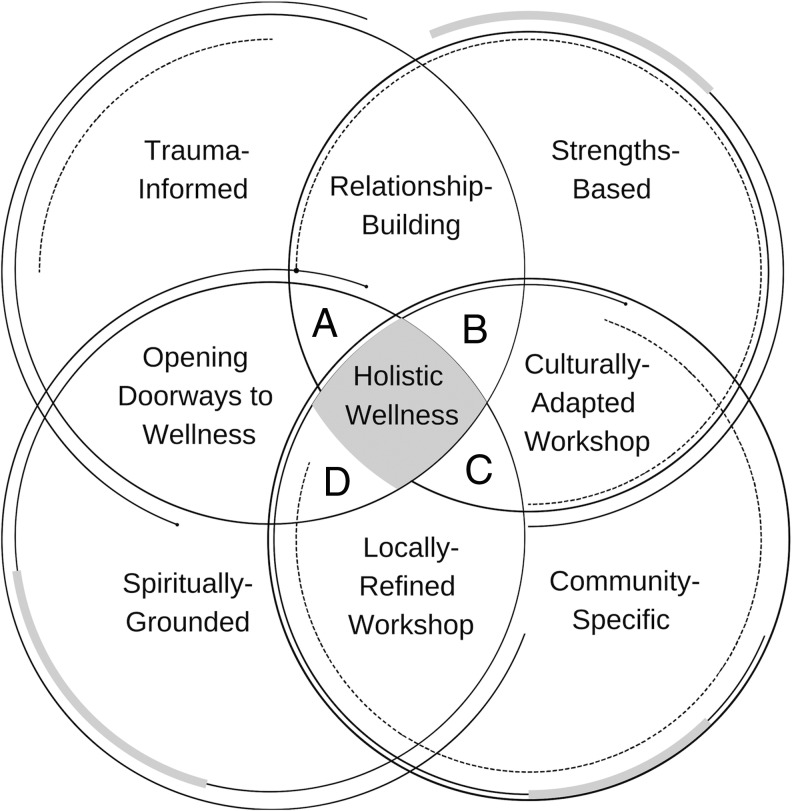
Our interrelated conceptual framework (circles), research stages (overlap), and coding layers **(A–D)** for Indigenous holistic wellness research.

The purpose of the animal-human relationship workshop was twofold. Firstly, it was used as an Indigenous *research method* to create the space for collective knowledge sharing of community-relevant animal stories (Absolon, 2011). Secondly, the curriculum itself (e.g., learning through animal-human relationship building activities) was used as an Indigenous *health promotion initiative* to facilitate cultural connectedness through the intergenerational process of remembering and (re)learning community-relevant stories of animal-human relationships that have been lost or weakened in the processes of colonization (Snowshoe et al., 2017). Our work was grounded in Snowshoe and Starblanket's (2016) conceptual framework, which addressed the need for more-than-human narratives to be acknowledged and woven through research and practice within the fields of social work, psychology, and environmental sustainability. Their framework was built on four “blankets” as metaphor, namely, (1) trauma-informed, (2) strengths-based, (3) community-specific, and (4) spiritually-grounded approaches that are critical for intergenerational healing to take place among Indigenous youth, families, and communities (see the four circles in Fig. 1). When “layered” or applied together, the blankets create a synergetic healing process for Indigenous peoples that can be applied equally well to research or therapy. This holistic approach to Indigenous wellness research emphasizes the need to understand Indigenous peoples' social realities within a larger context of colonization and intergenerational trauma, and to build upon the strengths that Indigenous peoples have demonstrated in the face of those traumas and cultural losses. It prioritizes the need to engage at individual, family, and community levels in processes of resilience-building by strengthening connections to culture and utilizing the agency of the more-than-human world as a doorway to holistic wellness.

The research process consisted of four highly interrelated stages, intertwining relational work, and the cultural adaptation and subsequent local refinement of a two-day animal-human relationship workshop that focused on teaching physical and spiritual methods for communicating with animals (refer to the overlap of circles in Fig. 1). At Stage One (Project Start: Relationship Building), a great deal of time was spent developing and strengthening research partnerships with the community using the relational and spiritual processes consistent with Indigenous and community-based participatory research, as described above. The community researchers, stakeholders, and traditional Elders from the community met with our group of researchers from the Universities of Regina and Saskatchewan to identify shared goals and mutual benefits from the research and to begin to co-create a two-day animal-human relationship workshop for the community. This unique workshop was culturally adapted from a basic (non-Indigenous) animal-human relationship workshop^1^. The development of the workshop ensured that the curriculum content, activities, and processes better supported cultural connectedness by privileging traditional ecological knowledge. It prioritized the storytelling method for knowledge co-creation (Archibald, 2008; Wilkins, 2004) and focused on bringing together First Nations youth and traditional Elders to facilitate intergenerational learning and sharing of stories about direct and indirect physical and spiritual communication with animals.

During Stage Two (Culturally Adapted Workshop: Gathering Stories), the workshop was implemented to begin the process of helping to (re)connect human participants with their more-than-human counterparts and gather data on that process. The research team collected workshop participants' stories of animal-human relationships via sharing circles, collective storytelling, individual interviews, feedback forms, researcher observations, and follow-up focus groups to obtain preliminary results regarding animals' roles in participants' lives and identify further refinements to the workshop that were needed from the perspective of the community.

Following the implementation of the culturally adapted animal-relationship workshop, Stage Three (Collective Analysis of Stories: Coming to Know) focused on preparing the data for collaborative analysis with the community, further strengthening our community-university collaborative capacity and skills, and deepening our collective understandings of the meanings of the animal-human relationship stories shared during the workshop. After significant preparation by the research team, the purpose for each of the workshop activities was collectively reviewed, and, through small- and large-group working sessions, traditional Elders and other community stakeholders proposed revisions which lay the theoretical, practical, and relational groundwork for a locally refined version of the workshop. The results of these collective analysis events informed the development of the locally refined animal-human relationship workshop and foregrounded traditional Elders' knowledge in identifying animals' roles for Indigenous holistic wellness.

Central to Stage Four (Locally Refined Workshop: Opening Doorways to Wellness) was the implementation of the locally refined animal-human relationship workshop and the subsequent development of an Indigenous wellness model based on the stories gathered from workshop participants. At this stage, there was a fundamental shift in the content and purpose of the workshop from remembering (learning) toward revitalizing (strengthening) locally relevant animal-human relationships as a health promotion initiative within the community. This was achieved through the addition of culturally-relevant and land-based components of the workshop that positioned more-than-human beings as amplifiers of powerful healing energy, and where workshop participants had more opportunity to utilize culturally-specific ways of relating to animals for healing and wellness purposes. The research team replicated the data collection approach from the preliminary workshop with the addition of a third day, in which participants created digital stories by using photographs and voiceovers to create and share narratives of how animals have had significant health-related impacts in their lives (Cunsolo Willox et al., 2013; Lambert, 2013).

## Data Analysis and Meaning-Making

The research team designed and utilized a “four-layered” approach to data analysis to align with our larger Indigenous research methodology and illustrate the interconnected relationships between animals and holistic wellness. Much of the work during the first two layers was completed by the university research team, while the community and Indigenous researchers were heavily involved in the third and fourth layers of analysis to drive the meaning-making process from an Indigenous epistemological and ontological perspective (Kovach, 2010; Walter and Andersen, 2013; refer to A through D, respectively, in Fig. 1). The first layer consisted of open coding, that is, coding the actual words offered by workshop participants (Saldaña, 2016). During the second layer, holistic and simultaneous coding methods were used to keep the stories authentic and “intact” (Lavallée, 2009; Saldaña, 2016). The third layer was used to rearrange/regroup the data into categories and subcategories (i.e., parent and child nodes, respectively, in NVivo 12) with a strong focus on Indigenous ways of knowing (i.e., spiritual and intuitive ways of knowing, Indigenous psychological concepts, etc.). The fourth layer focused on identifying linkages and relationships between Indigenous health-related theories (i.e., cultural connectedness) and to “tell the collective story.” The use of a situational analysis (mapping) technique (Clarke, 2005, 2012; Clarke, Friese, & Washburn, 2015; Kincaid & Fletcher, 2017) allowed for a blended Indigenous-qualitative approach to the analysis because of its visual ability to show connections and overlapping relationships. Situational mapping processes combined with insights from the more-than-human world were critical to connect categories of data into meaningful groups and reconnect the categories into a larger theoretical framework that explains the intersections between animals and holistic wellness from an Indigenous worldview. This multiple-perspective holism of Indigenous meaning-making consisted of “‘converging perspectives from different vantage points over time’ in real-life situations and settings (p. 24)” (Castellano, 2000, as cited in Lavallée, 2009, p. 22). It ensured that the data was synthesized and authentically integrated into an Indigenous worldview that made sense of the collective story and generated results that were culturally-respectful and relevant from the perspective of the community. The results of these analyses formed the core of the data represented in the results section below.

## Results and Stories

The following research findings are offered as an attempt to shed light on the role of animals for Indigenous holistic wellness, while prioritizing the need to keep the community stories intact. The following stories were selected based on their ability to capture the “essence” of the themes that emerged from the data set and should be considered Indigenous empirical knowledge in their own right; they are presented in a manner that leaves some space for individual interpretation, which is the strength and power of Indigenous storywork (Archibald, 2008). The results should not be viewed as a comprehensive model of the role of animals for Indigenous holistic wellness, but rather a glimpse into the importance of animals for a rural First Nations community in Saskatchewan that disrupts and challenges Western conceptions of health that tend to fragment understandings of holistic wellness for Indigenous peoples. The findings are not necessarily generalizable across the many Indigenous communities and Nations, but the larger worldview of connectedness to *all our relations* and its importance to physical, mental, emotional, and spiritual health is a transferable concept. Another potential for transferability is across the various communities in their shared fight to overcome similar health/wellness challenges caused by structural colonialism (Goodman & Gorski, 2014; Greenwood et al., 2015). A more-than-human perspective is a deeply rooted spiritual worldview shared across Indigenous cultures, and our results can arguably act as a thread among those diverse cultures.

### Animals in person roles

Extensive coding consisting of multiple and overlapping layers was completed, and the collective stories that emerged from the data identified both domestic and wild animals as playing many person roles, each defined below collectively by traditional Elder workshop participants^2^. These person roles are not metaphorical but rather assume all the sentience and agency that the term *person* implies. In fact, these more-than-human person roles have important implications for health, healing, and holistic wellness that were collectively verified by the community Elders and key stakeholders who had an extensive history with animals in this regard. Our collaborative data analysis revealed that animals act as messengers, providers, guides, helpers, teachers, protectors, and healers in the lives of Indigenous community members (for related work, see Dell, Chalmers, Dell, Sauve, & MacKinnon, 2008; Legge & Robinson, 2017; Snowshoe & Starblanket, 2016). These animal roles are highly interconnected. Our findings are consistent with the work by Legge and Robinson (2017) and further advance the understanding of animals in person roles through the identification of specific health-based impacts that come from strong animal-human relationships for Indigenous peoples. Furthermore, the results revealed that the power of animals in these various roles is further amplified when paired with culturally specific ways of relating with animals, specifically through the use of ceremony/prayer, traditional medicines/offerings, dreams/visions, language/song, and with the involvement of traditional Elders/knowledge keepers. Taken together, the results highlight our approach to strengthening animal-human relationships as a cultural connectedness and health promotion initiative.

#### Animals as messenger

Animals can physically and spiritually relay or transfer messages and information (i.e., through affective, visual, or auditory pathways). These messages can act as clues, signs, and/or warnings about something happening or about to happen, which can be positive or negative. Sometimes animals communicate with the spirits and with humans directly during physical interactions; at other times, they communicate indirectly through dreams, ceremonies, and traditional Elders. Animals and other more-than-human beings (e.g., rocks) can act as conduits and assist in conveying a message to another animal and to the Creator. An animal may come to humans all of a sudden, warning someone or bringing a message. Sometimes an individual will notice this happening because the animal is behaving oddly or something about the interaction is unusual or out of the ordinary. Occasionally, the message is not fully understood by the individual until after the fact but may become clearer with time, often with the assistance of a traditional Elder. These messages can prepare the receiver of the message physically (e.g., an approaching storm) or mentally (e.g., bracing for something) to lessen emotional pain.

Restoring balance and de-stressing:

This one time, I was really upset. We were at a funeral here, and the grandchildren I raised had gone to visit their mom and I was just worried. And there was this little blue bird that kept hovering around and here I spotted a nest. There's four little tiny, tiny eggs in that nest. And this one old guy came tapped me. He said, “That's your grandchildren; they're being taken care of.” You know, little signs like that, and he gave me a little feather, “This fell on your shoulder,” he said. “That's how I knew.” “Who gave this message to you?” I asked … I still have that little feather. I put it away because that was a message, that little tiny feather, a little blue one, fell on my shoulder. That's how that old man knew who to come and give that message to, that little messenger. (DT, Community Elder)

#### Animals as provider

Animals are considered providers of sustenance, energy, medicine, and teachings. When humans are in need of help, animals can provide it for us. For example, animals provide traditional medicines and teach humans where to find them (e.g., sage) by giving us information about where to find plants and other sources of food; they can physically or spiritually (e.g., via dreams) lead us to food and also directly provide humans and other animals with food (e.g., via hunting). Indirectly, certain animals (e.g., insects) act as providers by keeping other animals (e.g., birds) alive to feed humans. Animals also provide humans with practical tools (made from them) such as drums and clothing. In special circumstances, animals are considered as part of the family to the extent that they are referred to as a relative, sibling, or parent (e.g., clan systems). In these cases, this strong familial relationship prohibits the use of specific animals or animal parts of a particular species. In other cases, an inherent spiritual connection supports their traditional uses (e.g., bear grease). Because physical movement is necessary to connect with animals, they help humans acquire a better sense of balance, body awareness, coordination, flexibility, strength, cardiovascular conditioning, physical endurance, and stamina. Emotionally, animals provide companionship, compassion, and comfort to humans.

Provider of physical sustenance as food:

The boys there, me and [individual] there, we would always go hunt this one field by the river. It was really hard to get a deer out of there, but I dreamed that night, a bear came and talked to me and told me and showed me. He was telling me, this bear, he says, “You got to do it this way,” and sure enough we went to go hunt that field there and I told these boys here, “I had a dream last night,” I said, “and this is what that bear said, this is how we got to do it,” and sure enough, it worked, eh? We were finally able to get something out of there. (AG, Community Adult)

Communicating with animals, we did that when I was a kid with my Dad. He taught me how to understand an animal … he used to like going hunting ducks, whatever, and he taught me how to even whistle rabbits and the rabbits would come running. And he used to say, “What are you going to do with that rabbit? You want it for supper or are you going to leave it and let it go?” And we didn't need him so we let him go. Just like the rabbit understood the old man saying so we could kill him for supper. But he asked me first and nope, we just let it go. We will find something else. When you start doing this communicating with animals, that's the first thing that hit my mind. (JS, Community Elder)

Provider of traditional healing medicines:

I'm a medicine woman, and a lot of people come from all over come to get medicines. And so, a man comes in and he needed some bear grease. I just had a little bit in a little container, just had a little bit of bear grease … So I mixed his medicine in half of that. And I gave it to him, but I was feeling so cheap, eh. Like, “I can't give it all to you because what if an emergency comes. I have to have a little bit.” And he said, “That's okay,” he said. “Next time I'll come back, I'll bring you some,” he said. Okay fine. So, we did our trade, and it's a sweat [lodge] day. Go into the sweat, “Okay bear, *gwémé*. I need your help. You saw me. I was being cheap. I need your help. I need your oil, your grease, in order to help other people. I can't do it without your help. You have to share some of you with me.” That's how I prayed. So after the sweat, we get back … About twenty minutes later my ex-sister-in-law comes in … I served her my tea, and she had partly leftover feast food. She ate. She asked me, “Do you need bear grease?” She tells me, ‘“Do you need bear fat?” And I said, “Yes, I do need it really badly!” She said, “I have some. How about,” she said, “I bring it, you fix it, and you keep a share. I don't know how to fix bear fat.” … Here, meanwhile, I was just crying, eh. Thanking the bear. Like, when you ask and need, they supply. I was just being thankful to the bear for … *gwémé*. I guess, how do you say it in English? Namesake? I just thanked him. And so when you ask the animals for whatever you need, guaranteed, they will supply. And now, I just feel so honoured for having that gift: to be able to communicate. (DT, Community Elder)

Relaxation, peacefulness, and comfort:

I didn't really grow up with horses. Or, I've never really been around horses. So, I've always been, kind of scared, I guess you can say. And then when I went out there, and I just sat out there, and the horses are just walking around. It was really nice. And I wasn't scared. Yeah, it was very peaceful. Relaxing. (GB, Community Youth)

#### Animals as guide

Animals often act as the sources of guidance by helping humans with important life decisions. Animals are known to watch over and guide humans in practical ways (e.g., offering a perspective that provides insight) and spiritual ways (e.g., finding life purpose) throughout the life course. Animals also continue to guide humans after death and by helping individuals who have recently passed on to find their way during the transition from the natural world to the spirit world. For example, the horse or eagle might be present at a funeral to help reunite the deceased person with their relatives in the spirit world. Animals can guide humans directly or indirectly; their guidance can come through physical interactions during everyday activities and ceremonial processes, or through dreams and visions. Whether recognized by humans or not, animals are “always there” to provide constant support through their guidance. There are times when humans may ask to be guided by an animal; other times that guidance comes to someone unexpectedly.

Bereavement and grief:

My late father-in-law, he was kind of a simple old man. A lot of people thought that he was kind of close to being “not all there,” but he was. I don't know, the father in-law, like, he did everything with his heart. And I remember when we were putting him up when he had passed away, we were taking him up to the graveyard and we saw these horses lined up along the fence. They were dancing. They were dancing, and I asked [about this] in the ceremony. Next minute we looked around and the horses were all gone. There was this white horse—just a perfect dance to the beat of the drum—because we had the drums going up. And everybody saw these horses. There were quite a few of us that [said], “Did you see that?! Did you see that?!” And just a little further down the way, we looked; there were no horses there, and I asked in the ceremony if he was, this old man, a true traditional person because he was looked down upon all his life. He was kind of, he was [short pause], nobody took him serious, I'll say. But he worked for the spirits all the time, and the spirits came down and acknowledged this. They were taking him home. (DT, Community Elder)

Cultural identity, sense of self, and life purpose:

I just want to experience it. I want to experience a lot of the things these Elders talk about … Like those dreams and how it helps the Elders get through the day. What if one day I do actually dream of being with wolves? And then, it could change your life too, like it changes most of theirs, having those animals … I feel like it will have a big impact on me when I am older … Life is not all about staying indoors. There is something out there. It's pretty much just waiting for you to see them. (CS, Community Youth)

I'd like to share, just one story. It's not my story. It happened to my friend who I have come to see as a mentor. He was helping me along the way. He lives in B.C. and he was telling me that where they—I think he's Coast Salish—they have a spot in the forest where they usually go and do their offerings, and they'd tie flags and stuff after the ceremony. It's been used there for generations so they have hundreds of flags tied to the trees and that's where they did prayer and ceremony. But then this was a time when a wildfire happened and it burnt, that whole area that they used to go to for generations. When they went there, they seen that it was just ash and trees were burnt. The flags and stuff were burnt. They were really saddened because they lost all that history or that sentimental feeling of seeing the flags that had been used for many, many years. So they were very saddened and hurt, and they were trying to grasp what they were going to do next, and how they were going to recover from this kind of deal. Then, it was kind of like quiet, and then they started hearing these little tick tick tick all over the forest, and it sounded like rain almost. Until they kind of paid attention to what it really was, and what it was, is the squirrels actually in the forest were climbing up to the top of the trees and breaking off the pinecones and acorns and dropping them on the ground. And it was just showing them that's their way of just honouring the relationship and doing their work to help re-establish the natural process of rebuilding the forest. In turn, down the road, that they're going to be able to use that space again as a place to tie their flags up to the trees and stuff. So, it's just like a really powerful teaching tool that everyone, especially animals, have a purpose and they serve it just willingly, unknowingly even, of what's going to become of it. (FF, Community Adult)

#### Animals as helper

Animals act as partners by helping humans learn important life lessons (e.g., traditional teachings of love, humility, etc.). Their mere presence can help an individual feel supported in times of hardship (e.g., an eagle flies by and makes someone feel better). Humans may have a specific animal helper that was given to them at birth by a traditional Elder or in ceremony. Animals support mental wellness and mental preparation by helping to brace humans or prepare for what is to come. For example, horses have the sense to tell us how things are going to turn out, and the thunder spirit encourages individuals to pay attention and take note of the incoming weather. Ceremonies have certain cultural protocols, and animals have shown Indigenous peoples some of these steps to facilitate the power of ceremonial processes.

Animals help support sobriety:

I respect my animals. And, I really enjoy them. The friendship and companionship of my animals. Mine is that, I'm a recovering alcoholic. I used drugs. Mine is to say, the animals help me. For being there, talking to them. Sweat [lodge]. The rest here talk to the animals; I do the same thing. They don't talk the way we talk. They communicate it. And this is what helps me lots. (PR, Community Elder)

Animals help with physical ailments:

I always knew an eagle was my spirit helper, because about five years ago, I got very, very sick. I went up there twice and started going to sweats. And that's the first thing that would come by me in a sweat. I could feel it, a bear … I prayed in there, [the] night ceremonies. It would be an eagle that would come to me, and a bear holding my head. And I prayed so hard, I want to live some more, and see my *‘capans* [great-grandchildren], and here I am today. For four years, I kept getting sick, sick, almost the same time every year. And then, I got all doctored up. And this is the first thing that would come in there and, I don't know, bless me, or something. And then, I still go to night ceremonies, and thank the grandmothers, grandfathers, that looked after me to keep me still going. I will say, I will see my *‘capans* grow up. Even if I have to live to be a hundred [laughs]. Yeah, this is the one that always kept me going … I always knew the eagle was my spirit helper. And a bear, also, would always come in the sweat and the night ceremony. (VM, Community Elder)

#### Animals as teacher

Animals act as teachers by showing humans what to do through physical and spiritual interactions with them. Animals teach humans traditional values such as love, respect, truth, and humility. They also teach humans practical skills about how to care for ourselves and others (e.g., providing teachings to their young for humans to learn from). Animals constantly reinforce a cultural worldview through their instinctual interactions by showing humans how to live in collective harmony with one another. They teach humans to pay attention to signs (e.g., patterns of weather) about kinship (e.g., pack of wolves living together), how to be humble (e.g., bear and wolf restrain their strength), life lessons (e.g., herds protecting their young), and about collective betterment (i.e., treat everything as your relative). Different animal species offer certain teachings to us based on their intricate knowledge of and inherent relationship with the land. Because of their shared knowledge, we are able to “walk in a good way.”

Building resilience and accessing strength:

I have the four main guides: The bear, the eagle, the buffalo, and the wolf. And my power spirit is the hummingbird. And I draw my strength from them. Like, a lot of times, I'm am just so tired, “Okay guys, I need your help! I need that strength. I need that, ‘get up and go.’” And once you say it, even to think it, within the blink of an eye, they're there. You got your energy back … Everyday, no matter where I am, I'll always have to call on them. There's not a day that goes by that I don't draw on their strength, their power. Even in my lowest moments, when I'm feeling so defeated, “Hey!” and they're there to give me that jolt, that go-for-it attitude. (DT, Community Elder)

#### Animals as protector

Animals protect humans physically and spiritually by keeping us and our relations safe from danger (e.g., physical injury, illness, and bad medicine). They stay close to us to keep us safe or to get someone out of a dangerous situation (e.g., bears and wolves will walk beside someone during a long, dark journey home) and to intervene when negative energy is aimed at harming someone and/or their family or friends (e.g., horses are known to intercept bad medicine intended for family destruction). For example, if you are travelling and you strike a deer or an animal on the road, this could mean that they have intercepted something harmful that is further down the road so that you would not be involved or see what is up ahead (e.g., an accident); they delay you. Some animals come in ceremony to provide someone with spiritual protection, and children are known to have the ability to see these spirit animal protectors because they are still close to the Creator during this stage of their lives.

Animals provide physical protection:

I'll never forget about my spirit horse. If it wasn't for that spirit horse, I would be dead by now. Because that horse—I was laying—first I'll say that I was a little girl, maybe about ten, eleven, maybe younger, I don't know. I don't remember but it was the summer anyway—holiday from school. And it was a hot day and we had nothing to do. And then I just wanted to go in the shade and try and lay down there. Then all of a sudden when I was laying down in the shade there, I heard some noise [thumping], like some noise or something, like a trampling down. Then I saw this white horse, and there were a herd of horses in the back. And they came towards me. I sat up and that horse just stopped. Just stopped right there, maybe about six feet away. He looked at me. I looked at him. I didn't say anything, I was just shocked to see this horse here. I was scared. But he just looked at me. I saw his eyes. Then all of a sudden, after a while, just maybe trying to let me know something, I don't know, and I was just looking at him. Then all of a sudden, he made a grunting noise and all of a sudden they backed up. They didn't turn around right away. All of the horses backed up. And just quite a ways, that's when they turned around and started kicking and having trampled out of there, galloping out. And every morning, I got a picture of my grandson drew me. Drew me a picture of that horse, and we called it my spirit horse. Ever since that, every morning when I'm smudging, I'm thankful, thank the Creator, that I'm alive today because of that horse. (BG, Community Elder)

The horse has become my and my family's life and makes our whole family circle complete. We have a few dozen horses and they are the backbone of our family … In the mornings, we would find one horse [dead] and another morning we would another horse [dead], and I went into ceremony and I asked. That's when I found out that the horses intercepted all the bad medicines that were sent our way intending to harm my family because we had so many horses, jealousy, and whatever. The horses intercepted because of their love for us. And the respect that I have for the horse—when I found out, I was so, so grateful. I will never, ever talk bad about a horse ever again. Because in that ceremony, when I found out that the horse intercepted, I apologized. And the horse spirit come right by my shoulder and just told me he forgave me. Because I used to give my husband such a rough time for having so many horses. They'd get the best of what he had, they'd get the best food, even if we were short. He would always put his horses first. And after that, I would never say anything bad about the horse … And, you know, on the highway, we always see wild animals, wild game. They have been struck. We find them dead on the side of the road. Them too, they are interceptors. They always see ahead. (DT, Community Elder)

#### Animals as healer

Animals heal humans by providing love, compassion, and comfort. They heal by providing nutrients that help humans take care of ourselves (e.g., meat, honey, skunk oil). We can call animals for help (e.g., eagle, bear), and they show up. For example, some of us are physically healthy today because of animal and other more-than-human (e.g., grandfather and grandmother) healers. Mental peace of mind comes from knowing that the Creator's helpers have been sent to heal humans and that the illness is not coming back. Healers also provide emotional healing by providing comfort and someone to listen and take the pain away.

Animal intervention for mental illness and emotional disorders:

And, you know, when you're really depressed or going through something really hard, grab onto that. Doesn't matter where. Like, if you're going for a walk, walk. You'll see lots of rocks. But there's one that will pop out at you, like, you keep seeing it. Pick it up. It's meant for you. Pick it up. Draw on its power. Hold it in your hand. It'll carry you through. (DT, Community Elder)

Like a lot of times I've gone through life experiences that were really, really hard. I didn't trust telling any other human being. And this one old man told me a long time ago, he said, “Take your problems to a tree. Take them. Go offer tobacco to the tree, offer your print, and give it all you have, all the negative. It still stands tall and proud.” And you hear the leaves rustling, or the wind going through the leaves. That means it's taking all your problems away. You can cry. You can shout. You can give it your little details that you never, ever trusted another human being with. They'll take it all and they won't spread it. They take everything to the Creator and use it there. No other human being knows, just the tree, and it will stand firm and tall no matter how hard your experiences are—you've entrusted in his care. He'll still stand proud. (DT, Community Elder)

Literal intervention to treat and cure physical disease:

The eagle, that's who's helped doctor me from cancer four times. I've had arthritis, and whose claws got all that arthritis out from my joints, *kihew* and the bear. So I always carry those. These are my reminder. And you know, this white spot on my face? That's a reminder. I'm a survivor. Had cancer four times, and these guys helped me. That's my constant reminder. Every time I've looked at myself in the mirror, I see that. And I'm reminded, “Hey!” from where I came, where I should have been. That year, it was ’01, was the last time I had cancer, and it just about took me. I was not supposed to have seen fall that year, according to the doctors. But, with the Creator's help, and my spirit guides, my spirit helpers, I lived to tell. I'm a survivor. I survived cancer four times. Arthritis, that was supposed to have crippled me. And that day I got my wiggle back home, my god, I just—‘cause I couldn't, I couldn't move! I'd get locked from arthritis. And their claws dug it all out of my joints. (DT, Community Elder)

### Interrelationships

Situational analysis (mapping) highlighted the many interrelationships across animal roles and related health and wellness themes (i.e., the ways in which stories about animal roles intersected with stories about holistic health). For example, out of the 33 stories told during the animal-relationship workshops that were related to physical health in general, seven stories involved animals being responsible for curing serious physical disease (e.g., cancer), and 13 stories credited animals in the prevention of illness and physical injury. Interestingly, animals were most often acting in a protector role in these specific physical health cases. In regard to mental health (where a total of 49 stories were told), nine stories involved animals playing a key role in building resilience and accessing strength; three stories pertained to animals assisting in the treatment of emotional and clinical disorders (e.g., depression, anxiety); 10 stories referenced animals facilitating feelings of relaxation, peacefulness, and comfort (e.g., mindfulness); and five stories included animals aiding in restoring balance (between physical, mental, emotional, spiritual realms of the self) and de-stressing from difficult life circumstances. Animals in the helper role were found to be most influential for these positive mental health subcategories, with animals also playing a key healer role in many of these cases as well. Animals acted in the guide role most often for facilitating cultural identity, sense of self, and life purpose (from a total of 39 stories); a cohesive identity that extends into the future has been shown to be an important protective factor against Indigenous youth suicide (Chandler & Lalonde, 1998). The animal guide was also found to be important for assisting individuals suffering with grief and bereavement (from a total of eight stories; e.g., loss of a loved one to early death), some of which was related to cultural loss and its associated negative symptoms (see Armenta et al., 2016, for a review). Animals acting in a helper role were found in a total of 18 stories to be particularly important for those individuals struggling with a substance use issue, including discontinuing or decreasing substance use and maintaining sobriety.

It is important to recognize that animals assumed the various person roles simultaneously and in ways that have positive impacts across the broad and narrow (i.e., subcategory) health themes. Situational analysis enabled us to see a more holistic set of interconnections across animal roles and health-related themes that emerged from animal-human relationship stories. These connections between animals and holistic wellness are important to health practitioners because they emphasize the necessity of animal-human relationships from an Indigenous perspective while the specific interconnected stories provide examples that are meaningful at both individual and community levels.

## Conclusion and Implications

Indigenous constructs about the agency of animals provide valuable alternatives to commonly held Western medical and psychological perspectives, creating opportunities for deeper, more authentic relationships with animals that acknowledge and support their role in the healing process for Indigenous peoples. Health promotion and treatment initiatives that do not recognize and utilize Indigenous peoples' social relations with the more-than-human are bound to have limited effectiveness. Indigenous understandings of interwoven and reciprocal networks of human and more-than-human social relations must be better understood by health care service providers to remove oppressive systemic barriers that privilege Eurocentric treatment approaches and to facilitate Indigenous peoples' healing journeys. Reconciliation across Western and Indigenous contexts requires learning to work together with the more-than-human world, developing ethical spaces for health research in which wellness is appreciated and understood in the context of *all our relations*. In this paper, we provided concrete examples of how animals act as persons for Indigenous peoples and how they contribute to ways of healing. We call upon physicians, mental health practitioners, and educators alike to recognize, support, and incorporate these Indigenous knowledge systems into their practice and to carefully consider the ethical implications of not doing so in an increasingly culturally diverse society. Our findings provide an important “doorway” to Indigenous wellness and can be drawn upon in both development and implementation of health and education programs within individuals and group or community settings. The results provide applicable understandings of complex interconnected animal-human relationships that can aid in the development and implementation of health policy initiatives that flow from the findings shared in this article, with additional implications for public/postsecondary education, environmental sustainability and bioresource management, and youth justice.

## Footnotes

^1^ The basic animal-human relationship was facilitated by J. Kindred (https://www.intuitiveconnections.ca) building on some of the teachings of Williams (2003).

^2^ The research team gave all workshop participants the choice to use initials or pseudonyms associated with their stories during the informed consent process.
